# Evolutionary dynamics and differential mutation strategies of two avian pathogenic Escherichia coli lineages during experimental evolution

**DOI:** 10.1099/mgen.0.001893

**Published:** 2026-11-13

**Authors:** Yufei Zhao, Luo Yang, Dejun Liu, Jing Li, Henrik Christensen

**Affiliations:** 1 Department of Veterinary and Animal Sciences, University of Copenhagen, Copenhagen, Denmark; 2 College of Veterinary Medicine, China Agricultural University, Beijing, PR China

**Keywords:** biological fitness, cluster of orthologous groups, colibacillosis, fixation of beneficial mutations, nonsynonymous to synonymous substitution rates

## Abstract

Experimental evolution offers a powerful framework to investigate bacterial adaptation dynamics. In this study, we performed long-term evolution experiments over an estimated 376 generations under glucose-limited conditions using 2 clinical avian pathogenic *Escherichia coli* (APEC) lineages with strain 729-021014-1-4 representing sequence type 95 (ST95-729-021014-1-4) and strain 729-141114-1-15 representing ST131 (ST131-729-141114-1-15). Whole-genome sequencing of isolates across eight time points revealed distinct adaptive trajectories. The ST95-729-021014-1-4 lineage exhibited a rapid adaptation strategy characterized by a relatively high mutation accumulation rate. Sequence-based analysis predicted the transcriptional upregulation of the anti-adaptor protein IraP via a promoter mutation and the translational optimization of metabolic genes via synonymous codon changes as primary drivers of its improved growth rate. Conversely, the ST131-729-141114-1-15 lineage displayed a conservative evolutionary pattern with a lower mutation rate and minor growth rate improvement, suggesting a pre-adapted state that relied on genomic maintenance rather than extensive regulatory remodelling. Its mutations were largely restricted to envelope-associated genes and synonymous variants with reduced translational efficiency. While both lineages maintained clonal stability, their differential mutation strategies highlight lineage-specific potentials for adaptation. These findings provide critical insights into how different APEC clinical isolates may persist and evolve during the typical production cycle of poultry, underscoring the diversity of evolutionary strategies.

**Impact Statement**

Evolutionary dynamics of two avian pathogenic *Escherichia coli* lineages were observed and analysed under controlled laboratory conditions. While both lineages maintained overall clonal stability, they exhibited distinct trajectories in growth rate improvement and mutational patterns. During a typical production cycle of broilers, different wild-type clinical isolates may employ differential survival strategies, ranging from regulatory fine-tuning to conservative genomic maintenance, to adapt to new environmental stresses.

## Data Summary

The authors confirm that all supporting data and protocols have been provided within the article or through supplementary data files. All accession numbers are listed in the Supplementary Data Section.

## Introduction

Colibacillosis is a bacterial disease that significantly increases mortality rates in poultry flocks [1]. As the most prevalent bacterial infection in both layer and broiler chickens, colibacillosis affects farms worldwide, resulting in huge economic losses for the poultry industry and loss of animal welfare [2, 3]. The routes of avian pathogenic *Escherichia coli* (APEC) infection are from the intestinal tract and respiratory tract and ascending infection through the oviduct [4]. Colibacillosis commonly manifests as air sacculitis, cellulitis, peritonitis, salpingitis, swollen head syndrome, omphalitis, pericarditis or septicaemia or combinations of these [2, 5]. Notably, APEC shares significant genetic similarity with human ExPEC strains, particularly uropathogenic *E. coli*, raising concerns about its zoonotic potential [6, 7]. Multilocus sequence typing has been widely used to assign *E. coli* a numerical sequence type (ST) based on standardized schemes [8]. Clinical isolates of APEC have predominantly been identified as ST117, ST140, ST95 and ST131 [9]. For the current investigation, representatives of ST95 and ST131 were selected since they may also be isolated from extraintestinal infections in humans and have a potential for zoonotic transfer [10]. A representative strain for the current study was selected from ST95 since this ST has been reported from chronic chicken infections including outbreaks as well as different extraintestinal infection types in humans [2, 6, 10–12]. ST131 was first recorded with extended beta-lactamase resistance from human extraintestinal infections in the early 2000s [10] and later reported as APEC [12]. The two STs have a panel of potential virulence genes in common as well as large plasmids linked to virulence [13]; however, in other respects, ST95 and ST131 are divergent since they represent *E. coli* phylogroups B2 and A, respectively [2, 4].

Studies have highlighted the vertical transmission risk from broiler breeders with salpingitis to their progeny, and ST95 and ST131 and also other STs can cause first-week mortality under conventional production conditions [11, 12, 14].

Both epidemiological factors and inherent biological fitness have been suggested to contribute to the evolution and dissemination of APEC [10]. Applying an evolutionary investigation to APEC is crucial: intensive poultry production imposes strong, recurrent selection on virulence and transmission, driving patterns that translate into substantial welfare and economic burdens at flock scale. In parallel, APEC’s close genetic relatedness to human ExPEC creates a clear One Health interface; clarifying how APEC adapts within poultry populations can illuminate cross-host exposure pathways and guide targeted surveillance and control.

In bacteria, rapid evolutionary changes are evident, including adaptations for antibiotic resistance and slight improvements in fitness relative to their ancestors [15]. The most prominent long-term evolution experiment with *E. coli* is Richard Lenski’s long-term evolutionary experiment (LTEE), started in 1988. These experiments track 12 populations of *E. coli* over 80,000 generations in a glucose-limited medium, revealing insights into adaptation, fitness gains and genetic changes [16]. Prior LTEE studies demonstrated that fitness improvements were linked to changes in life-history traits, such as reduced lag phase and increased maximum growth rate, accompanied by phenotypic changes including larger cell size and modified metabolic profiles [17, 18]. In LTEE of *E. coli*, adaptive mutations predominantly have been found to occur early, with fewer beneficial mutations and a constraint of fitness improvement adaptation as populations approach local fitness peaks [19]. LTEE has been well established to illustrate the fitness improvement and adaptive mutation patterns of standard laboratory strains. Yet it is not known if results gained with laboratory strains can be transferred to environmental or clinical isolates of *E. coli* including APEC.

Here, we conducted a 2-month laboratory evolution experiment with strains representing some of the most important APEC lineages, ST95 and ST131. The aim of the study was to assess their clonal stability, identify potential adaptive mutations and deepen the understanding of the evolutionary dynamics and mechanisms of APEC in a simple glucose minimal medium model system.

## Methods

### Revival and adaptation of ancestor strains

Strain 729-021014-1-4 of ST95 (ST95-729-021014-1-4) and strain 729-141114-1-15 of ST131 (ST131-729-141114-1-15) of *E. coli* (Table 1) isolated and described in Poulsen *et al*. [14] were chosen as the ancestor strains for the evolution experiment. The strains were stored by freezing overnight-incubated broth cultures at −80 °C in 15% glycerol. The strains were cultured on blood agar [tryptose blood agar base (Difco, Brøndby, Denmark) added 5% bovine blood] overnight at 37 °C. A single colony of each ancestor strain was inoculated into 5 ml Luria–Bertani (LB) broth (BD Difco^™^, MD, USA) at 37 °C with rotation at 180 r.p.m. After 24 h, cultures were diluted 100-fold, and then, 100 µl of the diluted culture was transferred to 9.9 ml of M9 medium with 0.14 mM glucose. Minimal M9 medium was prepared from five times stock solution (Sigma Aldrich, M6030) diluted in sterile ion-exchanged water and added MgSO_4_, CaCl_2_ and thiamine to 200, 100 and 7.5 µM, respectively. Incubation was performed at 37 °C with rotation at 180 r.p.m. in 100 ml Erlenmeyer flasks.

**Table 1.** Strains investigated

Sample no.	Sampled date	Replicate no.	Days in experimental evolution	Approx. generations*
ST95 ancestor: 729-021014-1-4 (acc. no. CP160125)				
729_021014_1_4_19_1_24_1	2024.1.19	1	7	47
729_021014_1_4_19_1_24_2	2024.1.19	2		
729_021014_1_4_19_1_24_3	2024.1.19	3		
729_021014_1_4_26_1_24_1	2024.1.26	1	14	94
729_021014_1_4_26_1_24_2_	2024.1.26	2		
729_021014_1_4_26_1_24_3	2024.1.26	3		
729_021014_1_4_2_2_24_1	2024.2.2	1	21	141
729_021014_1_4_2_2_24_2	2024.2.2	2		
729_021014_1_4_2_2_24_3	2024.2.2	3		
729_021014_1_4_9_2_24_1	2024.2.9	1	28	188
729_021014_1_4_9_2_24_2	2024.2.9	2		
729_021014_1_4_9_2_24_3	2024.2.9	3		
729_021014_1_4_16_2_24_1	2024.2.16	1	35	235
729_021014_1_4_16_2_24_2	2024.2.16	2		
729_021014_1_4_16_2_24_3	2024.2.16	3		
729_021014_1_4_23_2_24_1	2024.2.23	1	42	282
729_021014_1_4_23_2_24_2	2024.2.23	2		
729_021014_1_4_23_2_24_3	2024.2.23	3		
729_021014_1_4_23_2_24_4	2024.2.23	4		
729_021014_1_4_1_3_24_1	2024.3.1	1	49	329
729_021014_1_4_1_3_24_2	2024.3.1	2		
729_021014_1_4_1_3_24_3	2024.3.1	3		
729_021014_1_4_1_3_24_4	2024.3.1	4		
729_021014_1_4_1_3_24_5	2024.3.1	5		
729_021014_1_4_8_3_24_1	2024.3.8	1	56	376
729_021014_1_4_8_3_24_2	2024.3.8	2		
729_021014_1_4_8_3_24_3	2024.3.8	3		
729_021014_1_4_8_3_24_4	2024.3.8	4		
729_021014_1_4_8_3_24_5	2024.3.8	5		
729_021014_1_4_8_3_24_6	2024.3.8	6		
729_021014_1_4_8_3_24_7	2024.3.8	7		
729_021014_1_4_8_3_24_8	2024.3.8	8		
ST131 ancestor: 729-141114-1-15 (acc. no. CP160108)				
729-141114-1-15_6.5_1	2024.6.5	1	7	47
729-141114-1-15_6.5_2	2024.6.5	2		
729-141114-1-15_6.5_3	2024.6.5	3		
729-141114-1-15_6.12_1	2024.6.12	1	14	94
729-141114-1-15_6.12_2	2024.6.12	2		
729-141114-1-15_6.12_3	2024.6.12	3		
729-141114-1-15_6.19_1	2024.6.19	1	21	141
729-141114-1-15_6.19_2	2024.6.19	2		
729-141114-1-15_6.19_3	2024.6.19	3		
729-141114-1-15_6.26_1	2024.6.26	1	28	188
729-141114-1-15_6.26_2	2024.6.26	2		
729-141114-1-15_6.26_3	2024.6.26	3		
729-141114-1-15_7.3_1	2024.7.3	1	35	235
729-141114-1-15_7.3_2	2024.7.3	2		
729-141114-1-15_7.3_3	2024.7.3	3		
729-141114-1-15_7.10_1	2024.7.10	1	42	282
729-141114-1-15_7.10_2	2024.7.10	2		
729-141114-1-15_7.10_3	2024.7.10	3		
729-141114-1-15_7.17_1	2024.7.17	1	49	329
729-141114-1-15_7.17_2	2024.7.17	2		
729-141114-1-15_7.17_3	2024.7.17	3		
729-141114-1-15_7.24_1	2024.7.24	1	56	376
729-141114-1-15_7.24_2	2024.7.24	2		
729-141114-1-15_7.24_3	2024.7.24	3		

*Assuming 6.7 generations per transfer [42].

### Evolutionary experiments

To initiate the experimental evolution, 100 µl of overnight culture for each lineage was transferred into 9.9 ml of fresh M9 minimal medium prewarmed to 37 °C and supplemented with 0.14 mM glucose and incubated at 37 °C with shaking at 180 r.p.m. for 24 h. After 24 h, growth was monitored by measuring the OD at 600 nm (OD₆₀₀) of a 1 ml culture sample. This was repeated every day for 56 days by transferring 100 µl of the 24 h culture to 9.9 ml of M9 minimal medium. To select isolates for archiving, 10 µl of culture was streaked on a blood agar plate every 7 days. From each plate, at least three single colonies were randomly selected and archived. For archiving, 438 µl of LB culture initiated from a single colony was mixed with sterile glycerol to a final concentration of 15% (v/v) in 1.5 ml cryovials and stored at −80 °C. The cultivation on blood agar was also used to verify the absence of contamination.

### Whole-genome sequencing

The archived strains originating from single colonies from different time points were cultivated on blood agar. Single colonies were cultivated in LB broth for DNA extraction. The DNA was extracted by Maxwell RSC Cultured Cells DNA kit (Promega, Madison, USA) according to the manufacturer’s instructions. The purity and concentration of extracted DNA were checked by NanoDrop (Thermo Fisher Scientific). The integrity of DNA was determined by gel electrophoresis. DNA libraries for Illumina sequencing were prepared using Illumina DNA Prep (Illumina, San Diego, CA), and sequencing reactions were prepared with Miseq Reagent kit v2 (Illumina, San Diego, CA). Samples were sequenced by the Illumina MiSeq platform using 2×300 bp paired-end sequencing strategy. Fastp v0.12.4 was used to remove adapters and filter short Illumina reads with default parameter settings [20]. Sequences were deposited with NCBI under BioProject number PRJNA1285664.

### Variant calling and mutation analysis

The annotated genomes of strains ST95-729-021014-1-4 (acc. no. CP160125) and ST131-729-141114-1-15 (acc. no. CP160108) were obtained from a previous study [13]. Breseq v0.39.0 [21] was used with ‘--consensus-minimum-total-coverage 20 j 8’ parameters to map trimmed reads to the GenBank format file of ancestral ST95-729-021014-1-4 and ST131-729-141114-1-15 genomes as references. Variant calling was performed for each time point. Gdtools embedded in the breseq pipeline was used to compare the presence and absence of mutations among sample reads using ‘gdtools COMPARE’ function as well as to count mutation types and numbers (with ‘gdtools COUNT’ function). The selection pressure acting on the evolving populations was assessed by calculating the ratio of nonsynonymous to synonymous substitution rates (dN/dS). The dN/dS ratio was computed using the following formula:


$$\frac{dN}{dS}=\frac{N_{obs}/N_{sites}}{S_{obs}/S_{sites}}$$

*N*_obs_ and *S*_obs_ refer to the number of nonsynonymous mutations and synonymous mutations. The theoretical ratio of synonymous to nonsynonymous sites (*S*_sites_/*N*_sites_) was estimated to be 0.315 based on the codon usage of *E. coli* coding sequences (CDSs) [22]. Mutation rate (*R*) based on gdtools COUNT was calculated as follows:


$$R=\frac{M}{N\times G}$$

*M* is the total number of single nt mutations, *N* is the generations and *G* is the reference genome size. Data visualization was performed using the R package ggplot2 (v4.0.1). The frequencies of mutated genes at one specific time point were estimated by calculating their average presence frequencies among all repetitive samples at that time point. Genotypes were inferred by grouping mutations with highly correlated temporal frequency trajectories. Hierarchical clustering was performed using the Pearson correlation coefficient as the similarity metric. Mutations exhibiting a correlation coefficient greater than 0.95 were assigned to the same genotype.

### Functional dynamics analysis

Cluster of orthologous groups (COGs) were annotated for each mutated gene with EggNOG mapper [23]. Synonymous mutations were included in this analysis to capture potential translational optimization effects. To visualize the temporal dynamics of mutation frequencies and their functional classifications, a comprehensive heatmap was constructed using the R package ComplexHeatmap (v2.26.0). The frequency of each identified mutation across all sampled generations was represented as a matrix, with intensity corresponding to the mutation frequency (scale: 0 to 1). Mutations were grouped and split vertically based on their COG functional categories.

### Transcriptional and translational regulation analysis

To investigate the potential regulatory impact of intergenic mutations, the promoter architecture was analysed *in silico*. A 100 bp sequence upstream of the start codon of the intergenic genes was extracted using SnapGene (GSL Biotech). The putative promoter elements (−10 and −35 boxes) and transcription start sites (TSSs) were predicted using the bacterial promoter prediction server BPROM [24]. To evaluate the effect of synonymous and nonsynonymous mutations on translational efficiency, the change in Codon Adaptation Index (ΔCAI) was calculated. The full CDSs of both the wild-type and mutated alleles were retrieved. The CAI values for each sequence were computed using the E-CAI server [25], where a reference set of highly expressed genes from *E. coli* K-12 was used.

### Protein structure modelling and stability analysis

To predict the structural impact of adaptive nonsynonymous mutations, comparative structural modelling was performed. The full amino acid sequences of both wild-type and mutant proteins were subjected to AlphaFold2 [26] to generate high-confidence 3D structural models. Structural alignment and visualization were conducted using UCSF ChimeraX [27]. Furthermore, the impact of single amino acid substitutions on protein thermodynamic stability was quantified regarding ΔΔ*G* using the PremPS server [28].

### Measurement of growth rate

To test changes in growth rate after 56 days, three single colonies of each ancestral strain and three strains from the last sampling of the experimental evolution were used as biological replicates. They were cultivated separately overnight at 37 °C on blood agar and then in 5 ml LB broth incubated overnight at 37 °C with rotation at 180 r.p.m. The following day, cultures were diluted 100-fold with physiological sterile salt water, and then, 1 ml of diluted cultures was transferred to 99 ml of M9 medium with 0.14 mM glucose. Growth curves were measured as OD at 600 nm (OD_600_) using 1 ml culture at each time point. The exponential phase was determined by selecting the period with the highest positive slope where the linear regression fit has *R*² >0.95. Growth curves were drawn in GraphPad Prism v11 software, and the growth rates (*μ*) were determined from the exponential growth phase as *μ* = (ΔlnOD_600_)/Δ*t*, where *t* is the time in hours and OD_600_ is the value determined at time *t* during exponential growth. A t-test was performed to compare the differences statistically between ancestral and experimentally evolved lineages within ST95 and ST131.

## Results

### Evolutionary adaptation and change in growth rate

To investigate the adaptative changes during the experimental evolution of clinical APEC, we selected ST95 strain ST95-729-021014-1-4 and ST131 strain ST131-729-141114-1-15. The two lineages were propagated individually for 56 days with a low concentration of glucose as the only carbohydrate source. Weekly, at least three single-colony isolates were archived, creating a longitudinal series of isolates that captured the evolutionary progression over time (Table 1). Adaptation was evaluated as growth rate increase. By the end of the evolution experiment, both lineages had adapted better compared to their ancestral strain according to the growth curves (Fig. 1). The initial average growth rate was 0.27 h⁻¹ (0.21±0.14) of the ST95 strain. After 56 days, the lineage was better adapted with an average growth rate of 0.71 h⁻¹ (0.59±0.20). The average growth rate of the ancestral ST131 strain was 0.60 h⁻¹ (0.55±0.11), and after 56 days, it had increased to 0.89 h⁻¹ (0.78±0.28) (Table S1, available in the online Supplementary Material). Significant increases in growth rates were observed among ancestral and evolved ST95 lineages (*P*<0.005, **) and ST131 lineages (*P*<0.05, *) (Fig. 2, Table S1).

**Fig. 1.**
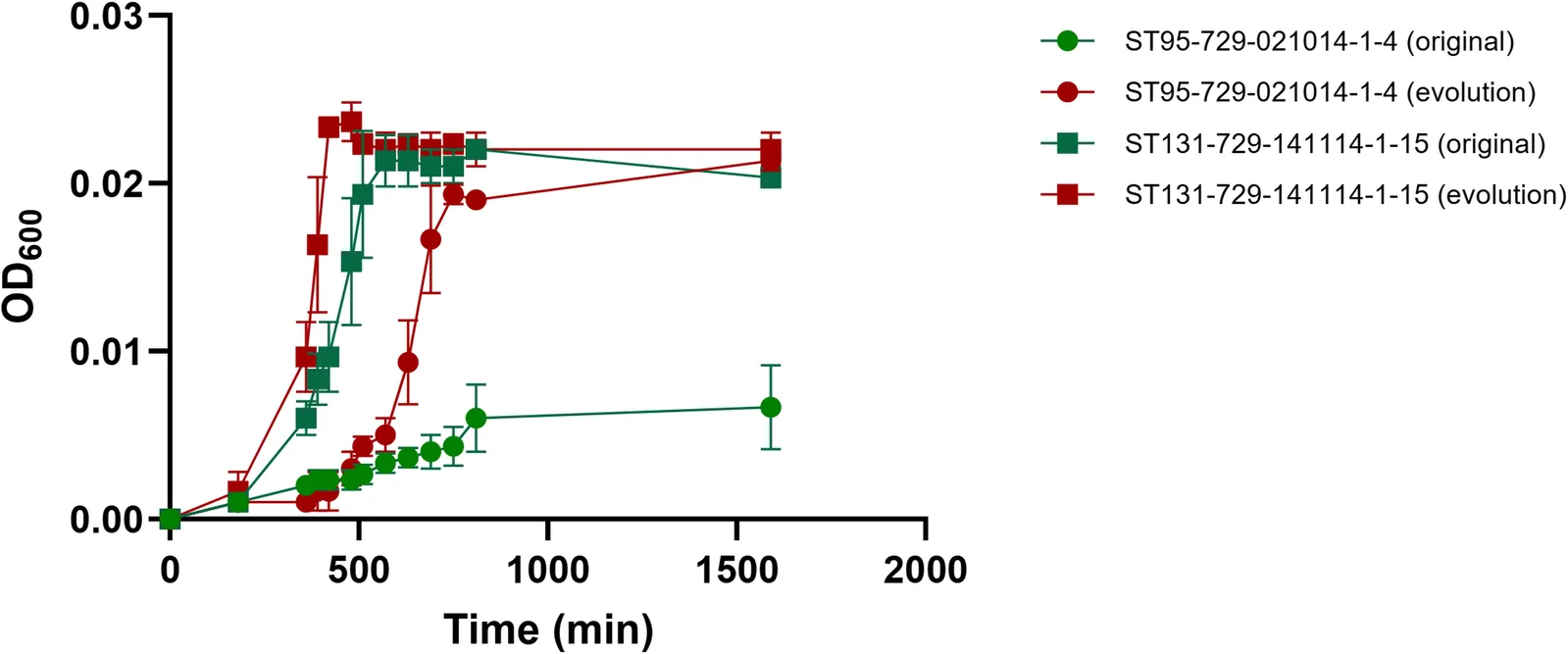
Growth curves of ST95-729-021014-1-4 and ST131-729-141114-1-15. Green curves are before and red after evolution for estimated 376 generations.

**Fig. 2.**
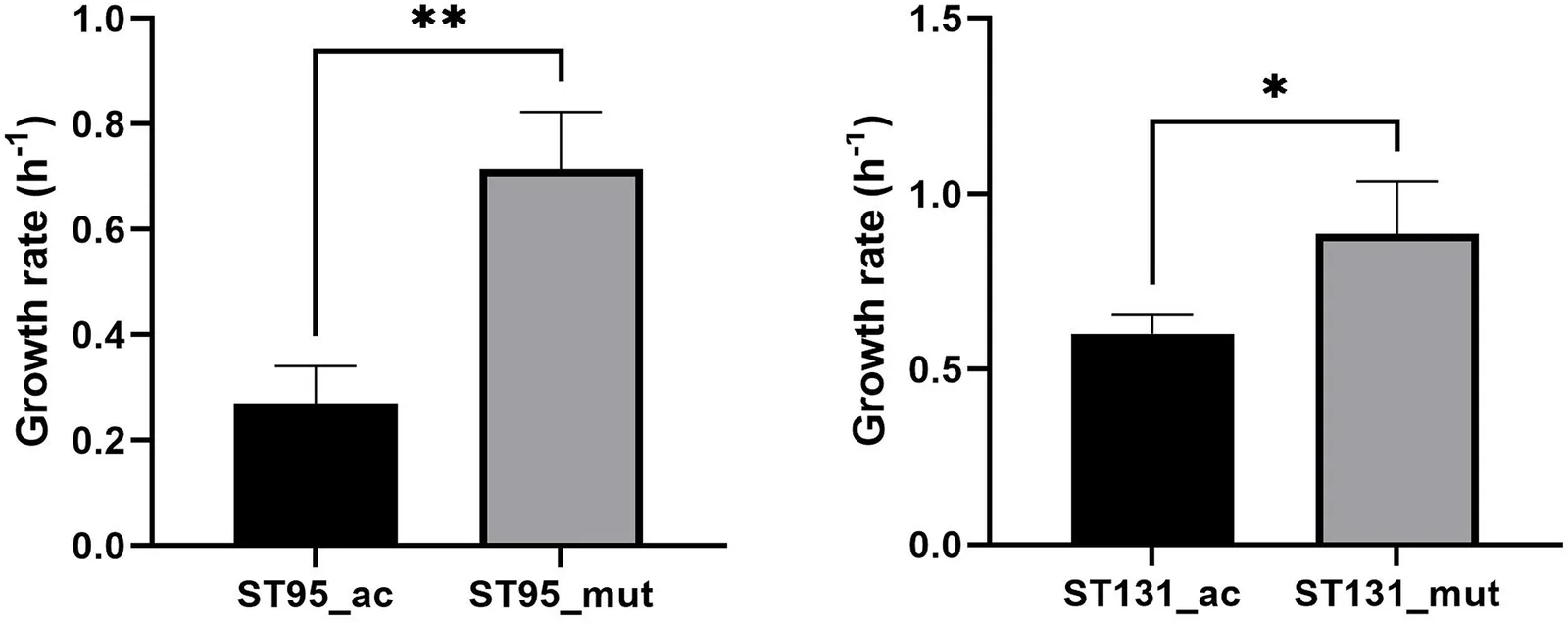
Growth rates of ST95-729-021014-1-4 and ST131-729-141114-1-15 lineages before (ac) and after (mut) evolution.

### Whole genomic sequencing statistics

For ST95-729-021014-1-4, a total of 32 isolates were sequenced during the experiment with daily transfer of cultures and collection of isolates. At least three isolates were selected for sequencing from each time point. The sequencing achieved an average coverage depth of 157×, ensuring sufficient resolution for downstream analyses. The overall sequencing quality was high with a Q20 rate of 97.01±0.43% and a Q30 rate of 92.88±0.87%, indicating reliable base-calling accuracy. For ST131-729-141114-1-15, a total of 24 isolates were sequenced with an average coverage of 411×. The sequencing quality was exceptionally consistent, with a Q20 rate of 99.34±0.02% and a Q30 rate of 97.65%, demonstrating robust read accuracy suitable for precise evolutionary comparisons.

### Overall mutational features

Generally, more mutations were found in the ST95-729-021014-1-4 lineage compared to ST131-729-141114-1-15 (Fig. 3). As shown in Fig. 3, base substitutions consistently account for the vast majority of detected genetic variations at each sampling point, increasing from 47 to 70 mutations from the beginning to the end of the experiment for ST95-729-021014-1-4. Small indels were present but maintained at low frequencies, while large structural variations were not detected. Synonymous mutations consistently outnumber nonsynonymous mutations. Throughout the experiment with an estimated 376 generations, the dN/dS ratio fluctuated within a narrow range of 0.19 to 0.25, providing strong evidence that the ST95 isolates are subject to strong purifying selection (Table S3). The mutation rate can be calculated as 3.23×10^−8^ per site per generation.

**Fig. 3.**
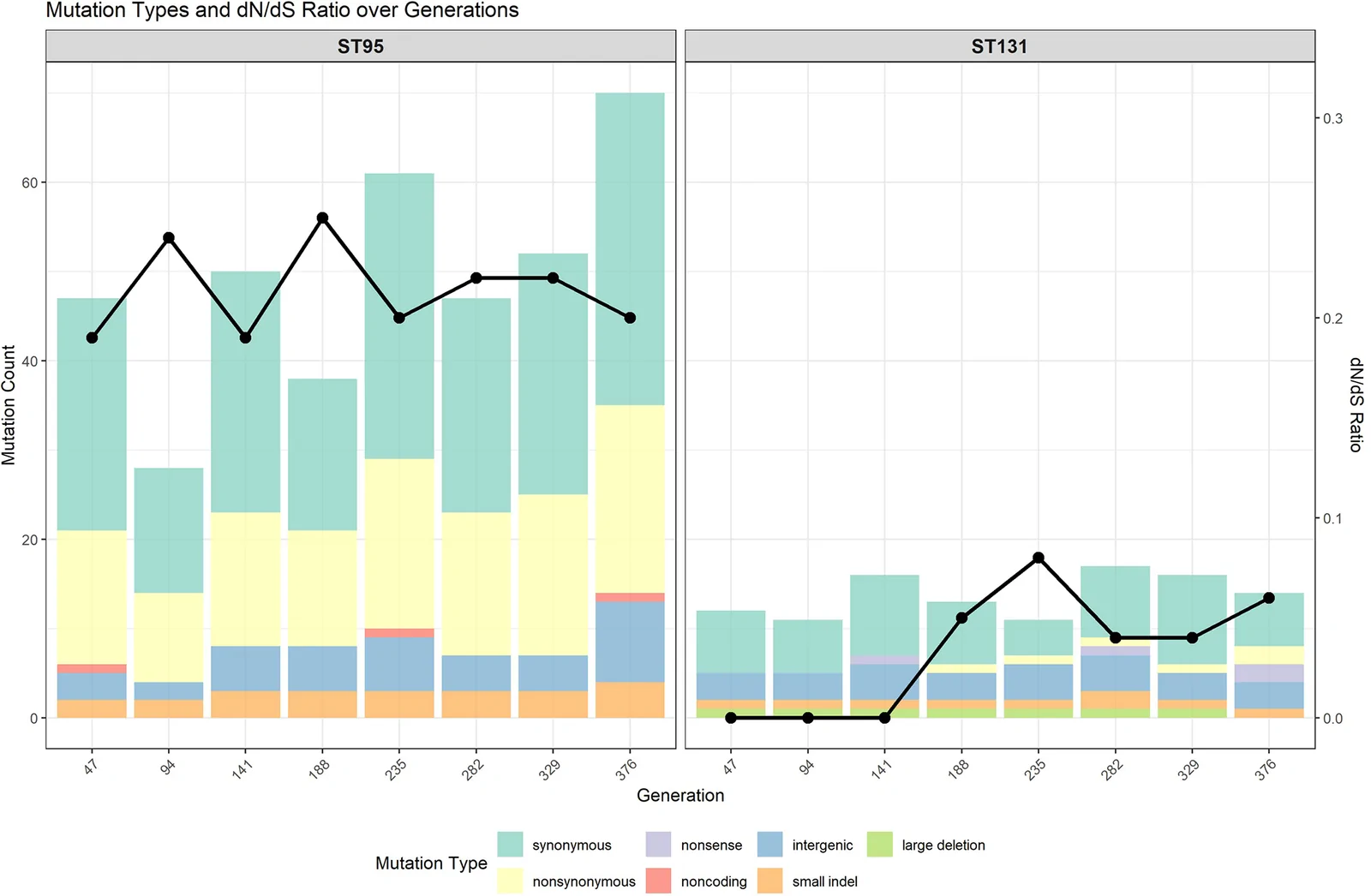
Overview of mutation types across all generations of ST95-729-021014-1-4 and ST131-729-141114-1-15 lineages after evolution. The number of mutations (counts) is shown on the left vertical axis. Black curves indicate the dN/dS ratio shown on the right vertical axis.

For ST131-729-141114-1-15, the number of mutations barely increased. A total of 14 mutations were identified across all lineages after an estimated 376 generations, spanning 5 genes and 3 intergenic regions. A large deletion was found across all generations except for the last sampling. Synonymous mutations consistently accounted for the vast majority of detected genetic variations in the descendants of the ST131 strain. For the first 150 generations, the dN/dS ratio remained at exactly 0. Then, the dN/dS rate exhibited an increase, rising from 0 to a maximum of 0.08, also indicating strong purifying selection (Table S4). The mutation rate was lower than that of the ST95 lineage, calculated as 6.15×10^−9^ per site per generation.

### Evolution trajectories of the two strains

For ST95-729-021014-1-4 lineages compared to the ancestral reference genome (acc. no. SAMN41822570), the founding mutation was identified as an intergenic C ->A transversion upstream of the *iraP* gene (Table S5). Promoter analysis mapped this mutation to the discriminator region, where the reduction in G+C content lowers the thermodynamic barrier for DNA melting, likely enhancing transcription initiation of the anti-adaptor protein IraP (Fig. S1). Consequently, this variant was designated as genotype *iraP+* and persisted throughout the evolutionary experiment. Eight additional mutations across five genes co-occurred with *iraP+* from the first sampling point, representing genetic hitchhikers. These included a single small indel and synonymous mutations. At the first sampling point at generation 47, the *iraP+* genotype was found in two out of three isolates analysed. Concurrently, a sublineage designated as genotype *klcA_T1*, which carried nonsynonymous mutations M85I and D91E in the antirestriction protein-encoded gene *klcA*, was found. This *klcA_T1* variant coexisted with the *iraP+* variant until it was eliminated after generation 188. Starting from generation 188, a nonsynonymous A15V mutation in another *klcA* gene copy emerged within the *iraP+* lineage and coincided with an I415V mutation in the phosphoethanolamine transferase-encoded gene *opgE*. This genotype was designated as *klcA_T2+opgE* and persisted in between one out of three isolates analysed at generation 188 and two out of eight isolates analysed at the last sampling. Additionally, genotype *vgrG* was detected starting at generation 235. This genotype was defined by four synonymous mutations including T73T, A77A, G78G and G80G in the actin cross-linking toxin-encoded gene *vgrG*. Besides, a nonsynonymous A403S mutation in the *glnD* gene encoding the bifunctional uridylyltransferase and a nonsynonymous A105P mutation in the *trmJ* gene encoding the tRNA methyltransferase, TrmJ appeared transiently but did not persist. A variant carrying a nonsynonymous N148K mutation in the *pykF* gene encoding pyruvate kinase I, along with a small indel in the *rpoS* gene encoding the RNA polymerase sigma factor RpoS, was detected at the 144th and 188th generations. At the final sampling time point, a variant was identified carrying a nonsynonymous T236P mutation in the *fumB* gene and a nonsynonymous F57L mutation in the *mgtA* gene encoding the magnesium-transporting ATPase; however, its potential for continued survival remains undetermined (Table S5).

For ST131-729-141114-1-15, compared to the ancestral reference genome (acc. no. SAMN41822574), a variant defined by two synonymous mutations, R378R and I376I, in the *cvaA* gene encoding the colicin V secretion protein CvaA appeared across all generations (Table S6). The frequency of this variant fluctuated throughout the experiment before ultimately sweeping the entire population at the end. A variant, characterized by a synonymous P377P mutation in the *leuC* gene encoding 3-isopropylmalate dehydratase, was observed throughout the evolution experiment but was completely eliminated by the end. Notably, a large 4,616 bp deletion spanning the region between the *ydhV* gene encoding for a putative oxidoreductase and the *pykF* gene encoding pyruvate kinase I consistently co-occurred with this *leuC* mutation. Within the *cvaA* lineage, a nonsynonymous Q273P mutation in the *uidC* gene encoding the membrane-associated protein occurred at generation 188. A nonsynonymous S315Y mutation in the *pykF* gene encoding pyruvate kinase I emerged at generation 282. Within the *leuC* lineage, a 2 bp deletion was identified in the *amiA* gene encoding the *N*-acetylmuramoyl-l-alanine amidase. All three of these genotypes were detected only transiently and did not persist in the population (Table S6). For these transient mutations observed, extra precautions should be taken about their occurrences related to few isolates sequences for each time point.

### Functional enrichment analysis

To elucidate the functional basis of the observed evolutionary trajectories, we mapped all identified mutations to COG categories. For ST95-729-021014-1-4, the heatmap revealed that adaptive mutations were not randomly distributed but clustered into distinct functional modules, predominantly metabolism (*n*=10) and cellular processes and signalling (*n*=9) (Fig. 4a). Within the metabolism category, mutations in genes related to ‘liquid transport’ (*atoB*), ‘inorganic ion transport’ (*ampG*, *mntB*) and ‘energy production and conversion’ (*fdrA*) maintained high frequencies throughout the experiment. Notably, many of these metabolic mutations were synonymous, suggesting that adaptive constraints on metabolism might be exerted at the translational level rather than through protein structural changes. In the cellular processes and signalling category, mutations were highly concentrated in signal transduction and membrane-associated genes (*opgE* and *vgrG*). Although *opgE* and *vgrG* defined distinct genotypes, their convergence within this functional category highlights its critical role in driving adaptation. The poorly characterized category was dominated by mutations on two different copies of the *klcA* gene (Table S7).

**Fig. 4.**
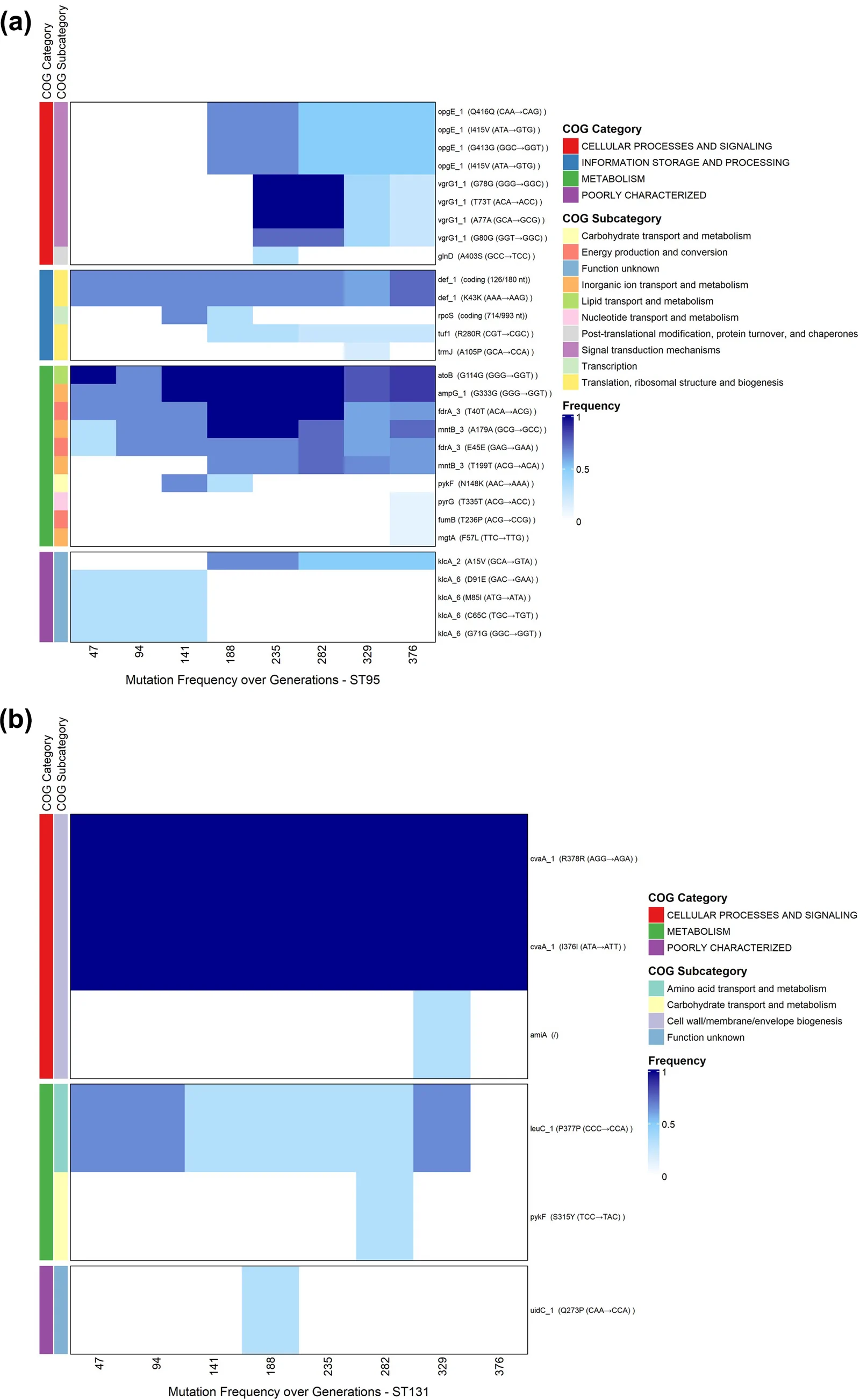
The COG annotations and grouping for representative mutation genes of ST95-729-021014-1-4 (**a**) and ST131-729-141114-1-15 (**b**) according to COG category. Frequencies of mutations are shown as heatmap, and the correlated gene names with their amino acid mutations are shown on the right of the heatmap.

For the ST131-729-141114-1-15 lineage, functional classification of the identified mutations revealed a streamlined landscape restricted to three primary COG categories (Fig. 4b). The cellular processes and signalling category was dominated by synonymous mutations in the *cvaA* gene which were further classified under ‘cell wall/membrane/envelope biogenesis’. As shown in the heatmap, this functional group maintained high frequency throughout the experiment and reached fixation. A transient 2 bp deletion in *amiA* was also mapped to this category. Within the metabolism category, mutations in genes related to ‘amino acid transport’ (*leuC*) were mainly identified. Transient mutation of *pykF* was related to ‘carbohydrate transport’. Additionally, the Poorly Characterized category consisted solely of the transient *uidC* mutation (Table S8).

### Translational regulation of mutated genes

To evaluate whether the observed mutations contributed to adaptive fitness through translational regulation, the change in ΔCAI was calculated for all mutated genes. In the ST95-729-021014-1-4 lineage, a widespread trend towards codon optimization was evident. Synonymous mutations defining the *vgrG* lineage (maximum ΔCAI=0.005) and those in metabolic genes such as *atoB* (ΔCAI=0.001), *ampG* (ΔCAI=0.003) and *mntB* (ΔCAI=0.002) predominantly exhibited positive ΔCAI values, suggesting systematic selection for enhanced translation elongation efficiency. This optimization trend extended to nonsynonymous mutations; notably, the fixed I415V mutation in *opgE* displayed the highest increase in ΔCAI up to 0.008 among all detected variants, indicating a potential synergistic effect where the mutation confers advantages through both structural modification and translational maximization. In contrast to this general trend, mutations within the *klcA* gene consistently exhibited negative ΔCAI values across different mutation types. This recurrent introduction of rare codons in *klcA* implies a specific evolutionary constraint (Table 2). The ST131-729-141114-1-15 lineage exhibited a distinct evolutionary pattern regarding codon usage. The dominant synonymous mutations in *cvaA* (R378R, ΔCAI=−0.004; I376I, ΔCAI=−0.005) and *leuC* (P377P, ΔCAI=−0.005) were associated with a reduction in ΔCAI (Table 2).

**Table 2.** CAI* for mutations

Gene	Annotation	CAI_Wild-Type_	CAI_mutant_	ΔCAI
ST95				
*fumB*	T236P (ACG→CCG)	0.685	0.691	0.006
*opgE*	G413G (GGC→GGT)	0.679	0.682	0.003
	I415V (ATA→GTG)	0.679	0.687	0.008
	Q416Q (CAA→CAG)	0.679	0.685	0.006
*mgtA*	F57L (TTC→TTG)	0.673	0.679	0.006
*klcA*_T2	A15V (GCA→GTA)	0.721	0.715	−0.006
g*lnD*	A403S (GCC→TCC)	0.673	0.681	0.008
*vgrG*	T73T (ACA→ACC)	0.698	0.703	0.005
	A77A (GCA→GCG)	0.698	0.699	0.001
	G78G (GGG→GGC)	0.698	0.697	−0.001
	G80G (GGT→GGC)	0.698	0.699	0.001
*ampG*	G333G (GGG→GGT)	0.661	0.664	0.003
*mntB*	T199T (ACG→ACA)	0.668	0.670	0.002
	A179A (GCG→GCC)	0.668	0.676	0.008
*pykF*	N148K (AAC→AAA)	0.680	0.776	−0.004
*atoB*	G114G (GGG→GGT)	0.665	0.666	0.001
*t*rmJ	A105P (GCA→CCA)	0.689	0.684	−0.005
*pyrG*	T335T (ACG→ACC)	0.680	0.680	0
def	K43K (AAA→AAG)	0.664	0.666	0.002
*tuf*1	R280R (CGT→CGC)	0.660	0.660	0
*klcA*_T1	C65C (TGC→TGT)	0.724	0.717	−0.007
	G71G (GGC→GGT)	0.724	0.715	−0.009
	E45E (GAG→GAA)	0.724	0.720	−0.004
	T40T (ACA→ACG)	0.724	0.719	−0.005
*fdrA*	E45E (GAG→GAA)	0.681	0.684	0.003
	T40T (ACA→ACG)	0.681	0.681	0
ST131				
*leuC*	P377P (CCC→CCA)	0.684	0.679	−0.005
*uidC*	Q273P (CAA→CCA)	0.670	0.670	0
*pykF*	S315Y (TCC→TAC)	0.678	0.680	0.002
*cvaA*	R378R (AGG→AGA)	0.672	0.668	−0.004
	I376I (ATA→ATT)	0.672	0.667	−0.005

*E. coli K12 codon usage table: UUU 22.4 (20462) UCU 8.5 (7788) UAU 16.2 (14828) UGU 5.2 (4731) UUC 16.6 (15160) UCC 8.6 (7879) UAC 12.2 (11181) UGC 6.4 (5882) UUA 13.9 (12726) UCA 7.1 (6481) UAA 2.0 (1830) UGA 1.1 (976) UUG 13.7 (12532) UCG 8.9 (8162) UAG 0.3 (286) UGG 15.3 (13993) CUU 11.0 (10079) CCU 7.0 (6427) CAU 12.9 (11776) CGU 20.9 (19089) CUC 11.1 (10115) CCC 5.5 (5004) CAC 9.7 (8869) CGC 22.0 (20120) CUA 3.9 (3569) CCA 8.4 (7711) CAA 15.2 (13910) CGA 3.6 (3304) CUG 52.8 (48324) CCG 23.2 (21245) CAG 29.0 (26522) CGG 5.4 (4970) AUU 30.4 (27828) ACU 8.9 (8140) AAU 17.8 (16259) AGU 8.8 (8033) AUC 25.0 (22880) ACC 23.4 (21403) AAC 21.6 (19782) AGC 16.0 (14644) AUA 4.4 (3998) ACA 7.1 (6465) AAA 33.6 (30739) AGA 2.1 (1953) AUG 27.8 (25442) ACG 14.4 (13186) AAG 10.3 (9463) AGG 1.2 (1096) GUU 18.3 (16766) GCU 15.2 (13955) GAU 32.2 (29468) GGU 24.7 (22591) GUC 15.3 (14002) GCC 25.5 (23336) GAC 19.1 (17482) GGC 29.6 (27072) GUA 10.9 (9967) GCA 20.3 (18585) GAA 39.5 (36159) GGA 7.9 (7224) GUG 26.3 (24080) GCG 33.7 (30843) GAG 17.8 (16301) GGG 11.0 (10041) where the decimal number is frequency out of 1,000 and the number in parentheses the total count per genome.

### Analysis of protein structures

To elucidate the molecular mechanisms driving the fixation of the *klcA_T2+opgE* variant at the end of the experiment, we analysed the structural and thermodynamic impact of its defining nonsynonymous mutations. This variant is characterized by an A15V mutation in the antirestriction protein KlcA and an I415V substitution in the phosphoethanolamine transferase OpgE. Structural modelling of KlcA positioned the A15V mutation at the critical hinge region connecting the flexible N-terminal tail to the structured globular domain (Fig. S1). The substitution of the small alanine residue with the bulkier valine side chain increases local packing density and likely restricts the conformational entropy of the flexible tail. Thermodynamic analysis yielded a folding free energy change of −0.5 kcal mol^−1^, classifying A15V as a stabilizing mutation (Fig. S1). Structural modelling of OpgE using AlphaFold did not reveal any obvious destabilizing effects or disruption of domain architecture associated with the I415V mutation. Consistent with this structural observation, thermodynamic analysis yielded a ΔΔ*G* of 0.2 kcal mol^−1^, which falls below the standard threshold (ΔΔ*G*>0.5 kcal mol^−1^) typically indicative of significant stability changes.

## Discussion

Conventional fast-growing broilers typically reach slaughter weight at ~35 days, whereas slower-growing broilers use ~84 days [29]. Our 56-day APEC experimental evolution design was a compromise to simulate the production time in the two production systems, assuming that natural populations undergo regular cycles of growth and stationary phase related to nutrient availability or limitations, respectively.

During the 56-day evolution experiment, the ST131-729-141114-1-15 lineage exhibited a relatively higher growth rate and clonal stability compared to ST95-729-021014-1-4. According to the growth rate, initially, ST95-729-021014-1-4’s growth rate was significantly lower than that of ST131-729-141114-1-15, but by the end of the evolution experiment, the growth rate of both lineages converged somehow. This convergence may result from constraints imposed by reaching local fitness peaks or could be due to growth limitations caused by nutrient restrictions in the culture medium. Notably, in Lenski *et al*.’s study, the maximum OD (OD_600_) of *E. coli* in LTEE at generations 76,000–76,500 in DM25 medium was measured as 0.0252 (range: 0.0239–0.0265) [30]. This is consistent with our results for both lineages after the estimated 376 generations of evolution in M9 medium, where the maximum OD_600_ was ~0.025.

We can find interesting patterns of subtle mutations for these two lineages probably related to their divergent fitness. For ST95-729-021014-1-4, *iraP+* was the first dominant genotype which swept out genotype *klcA_T1* in 188 generations. IraP is a small anti-adaptor protein that directly interferes with this process by binding to response regulator adaptor RssB and preventing the delivery of the sigma factor RpoS to the ATP-dependent protease ClpXP, thereby stabilizing RpoS at the post-translational level (Fig. 5) [31]. In *E. coli*, RpoS (σ^S) is an alternative sigma factor that serves as the master regulator of the general stress response, enabling the bacterium to survive a wide range of stresses such as nutrient starvation, osmotic stress and oxidative stress [32]. It plays a central role in adaptation to many suboptimal growth conditions by controlling numerous genes that protect the cell from stress and help scavenge nutrients [33]. In the short term of the LTEE, mutations that reduce RpoS activity can provide a selective advantage by reallocating resources from stress resistance to growth, whereas over thousands of generations such RpoS-attenuating mutations tend to become fixed, reflecting an evolutionary trade-off that favours enhanced nutrient acquisition at the expense of stress tolerance in a stable environment [34]. Expression of the gene *iraP* is strongly induced under phosphate starvation and is positively regulated by the alarmone ppGpp, linking nutrient stress sensing to RpoS stabilization [35]. Increased *iraP* transcription under nutrient-limited conditions provides a route for elevating RpoS protein levels without altering *rpoS* transcription, allowing cells to rapidly activate the RpoS regulon in response to environmental stress. This coupling of nutrient-stress signalling with RpoS stabilization also indicates that evolutionary selection pressures are acting at the stationary-phase stage of growth, favouring traits that enhance survival under prolonged nutrient limitation [33].

**Fig. 5.**
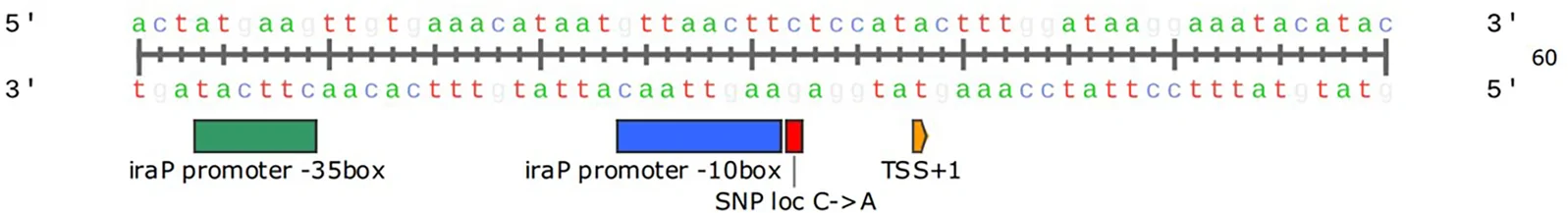
The intergenic mutation between *ddlA* and *iraP* happened specifically in the discriminator region of *iraP*, located between the −10 hexamer and the TSS. The C ->A transversion substitutes was predicted to increase the transcription efficiency of *iraP*. Increased expression of the *iraP* gene can upregulate *rpoS* gene, which is essential for the growth of *E. coli* in stress condition.

During the course of evolution for ST95-729-021014-1-4, two genotypes emerged subsequently and remained fixed until the end of the growth experiment: one driven by the nonsynonymous mutations in KclA and OpgE and another driven by the synonymous mutation in VgrG. Although KclA mutations have not been linked to fitness effects in the literature, available evidence for OpgE indicates a pivotal role in osmoregulated periplasmic glucan biosynthesis, which is known to influence osmotic adaptation, motility and biofilm formation via modulation of the Rcs phosphorelay system [36]. Additionally, VgrG proteins serve as structural components of the type VI secretion system, instrumental for interbacterial competition and effector delivery. Mutations in VgrG have been shown to alter competitive fitness in several Gram-negative bacterial species [37, 38]. Besides, several non-synonymous mutations in transposons and hypothetical proteins were found. Although they were excluded on evolution trajectory maps, they may still influence fitness via indirect or regulatory mechanisms. In an experimental study using Tn10 mutagenesis, environment-specific analysis showed that some insertions in random genes were beneficial to bacterial fitness [39]. Hypothetical proteins are often annotated as genes of unknown function, but experimental evolution studies revealed that loss-of-function mutations (often in nonessential or uncharacterized genes) can alter fitness landscapes via epistatic and environment-dependent effects [40].

For ST131-729-141114-1-15, only two sites on the *cvaA* gene underwent synonymous mutations, with no other genotype becoming fixed. Currently, no studies have linked mutations in the *cvaA* gene to changes in fitness. At the 282^th^ generation, a non-synonymous mutation in *pykF* was detected, representing the only overlapping mutated gene between ST95-729-021014-1-4 and ST131-729-141114-1-15. In the LTEE of *E. coli*, adaptive mutations predominantly occur early, with fewer beneficial mutations and constraints on fitness improvement as populations approach local fitness peaks [19]. Although our experiment spanned only 378 generations, we observed a similar phenomenon, supporting the notion that the ST131-729-141114-1-15 lineage exhibited a superior growth rate and clonal stability compared to ST95-729-021014-1-4 even prior to the evolution experiment.

Under glucose limitation, lineage ST95-729-021014-1-4 accumulated numerous mutations in metabolic pathways, a classic adaptive strategy to maximize carbon acquisition and energy efficiency. The enrichment of mutations in inorganic ion transport (*mgtA*, *mntB*) and energy production (*fumB*, *fdrA*) suggests optimization of nutrient uptake and central metabolism to cope with glucose scarcity. This metabolic reprogramming, including recurrent mutations in genes like *fdrA*, *atoB*, *mntB* and *ampG*, is well documented in *E. coli* evolution experiments where parallel mutations improve growth yield on limited glucose [41, 42]. The prevalence of these mutations aligns with findings that nutrient limitation favours alterations that redirect cellular resources towards growth [43]. Additionally, mutations in ‘cellular processes and signalling’ indicate that restructuring cellular machinery, possibly to reduce maintenance costs or alter permeability, also contributed to adaptation [44]. This mutational profile illustrates a broad physiological adjustment consistent with known *E. coli* responses to long-term nutrient limitation.

In contrast, lineage ST131-729-141114-1-15 evolved via a narrower set of mutations, primarily targeting cell envelope-related ‘cellular processes’. Two independent mutations in *cvaA* suggest strong selection to abolish this costly bacteriocin secretion system. Disabling energetically expensive functions, such as colicin production, confers a growth advantage when nutrients are scarce and competition is absent, a phenomenon observed in laboratory-evolved bacteria that shed unnecessary virulence or plasmid-encoded factors to streamline survival [44, 45]. Thus, reducing cell envelope energy expenditure was likely a key adaptation. Few metabolic mutations emerged, except for a notable late-arising nonsynonymous mutation in *pykF*, which adjusts glycolytic flux and improves efficiency [41, 46]. This trajectory highlights a distinct adaptive route: conserving resources by discarding expendable functions and focusing on core metabolism, which aligns with known patterns of *E. coli* evolution under nutrient limitation [45, 46].

Clonal stability may reflect either the competitive advantage of certain clones or a low mutation rate. The ST131 lineage displays stronger clonal stability than ST95, which is also evident from the number and types of mutations. After the estimated 376 generations, lineage ST95-729-021014-1-4 accumulated a total of 70 mutations, including 21 non-synonymous changes. According to COG enrichment analysis, the most affected functional categories were metabolism (*n*=9). Lineage ST131-729-141114-1-15 acquired only 14 mutations, of which only 1 was non-synonymous, and it also featured a large deletion. Its most impacted category was cellular processes and signalling (*n*=2). Given that the evolution took place under low-glucose, nutrient-limited conditions, the focus on metabolic gene mutations in ST95-729-021014-1-4 indicates effective environmental selection driving growth rate improvements. In contrast, lineage ST131-729-141114-1-15 experienced much weaker selection pressure.

The mutation rate of wild-type K-12 *E. coli* MG1655 is 2.2×10⁻¹⁰ mutations per nt per generation [47]. This is consistent with our previous estimates for ancestral ST95-729-021014-1-4 (2.87×10⁻⁷ SNPs per site per year, equivalent to 1.17×10⁻¹⁰ SNPs per bp per generation) and ST131-729-141114-1-15 (7.33×10⁻⁷ SNPs per site per year, equivalent to 3.00×10⁻¹⁰ SNPs per bp per generation) in natural condition [13]. However, in the experimental condition, the accumulated mutation rate for ST95-729-021014-1-4 was calculated as 3.23×10⁻⁸ per site per generation, while for ST131-729-141114-1-15, it was 6.15×10⁻⁹ per site per generation. The elevated mutation rate observed in our glucose-limited laboratory populations is consistent with stress-induced mutagenesis in *E. coli*. Nutrient starvation can drive a several-fold increase in mutation rates compared to nutrient-rich conditions. Meanwhile, although the mutation rate was high, the dN/dS ratio was low, which usually means purifying selection. As shown in our results, synonymous mutations affect the adaptation of ST95-729-021014-1-4 by increasing transcription via the *iraP* promoter and optimizing translation efficiency in several genes. This matches previous findings that synonymous mutations can help protein folding or expression levels without breaking protein structure [48]. On the other hand, not many mutations were found in ST131-729-141114-1-15. This indicates that the two involved lineages adapted to the evolutionary environment in different ways, even though they all adapted to the experimental environment somehow.

### Conclusions and perspectives

In this study, we characterized the evolutionary dynamics of two clinical APEC lineages, ST95-729-021014-1-4 and ST131-729-141114-1-15, under controlled laboratory conditions. The strains belonged to clones associated with widespread and serious disease in both humans and poultry. The ST95 strain was predicted by whole-genome sequence comparison to belong to serotype O2/O50:H5 and fimbria type H526, and the ST131 strain was predicted to be serotype O25:H4, clade B and fimbria type H22 in a previous study [13]. While both lineages maintained overall clonal stability, they exhibited distinct trajectories in growth rate improvement and mutational patterns, reflecting differential adaptive strategies. The ST95-729-021014-1-4 lineage demonstrated a strategy of rapid adaptation driven by a high mutation accumulation rate. Its evolution was characterized by the swift fixation of beneficial mutations, primarily through the transcriptional upregulation of the stress-response regulator *iraP* and the translational optimization of metabolic genes via synonymous codon changes. In previous investigations, elevated mutation rates have also been observed in the strains of ST95 [11]. In contrast, the ST131-729-141114-1-15 lineage displayed a more conservative evolutionary pattern, characterized by a lower mutation rate and a slower increase in growth rate. None of the typical potential virulence factor genes of APEC were detected among genes affected by non-synonymous mutations or indels, and rather, the genes affected (*atoB*, *fumB*, *glnD*, *iraP* promotor, *klcA*, *mgtA*, *mntB*, *opgE*, *pykF*, *rpoS*, *trmJ*, *uidC* and *ydhV*) were involved in core metabolic functions (*n*=7) and cellular processing and signalling processes (*n*=5) of *E. coli* such as nitrogen source sensing, metal ion transport, adaptation to anaerobic conditions and general stress response showing the high capacity of APEC to adapt to its environment. It should be noted that this study focused primarily on single nt variants. The role of structural variations, such as insertions, deletions and large-scale chromosomal rearrangements mediated by mobile genetic elements, warrants further investigation. Future studies utilizing hybrid assembly strategies, combining short-read Illumina and long-read Nanopore sequencing, will be essential to fully resolve these complex genomic events and provide a comprehensive understanding of APEC genome evolution. LTEE performed in glucose minimal medium is the most basic model system to estimate mutations in APEC. Further investigations using this model will be needed to test one by one a range of stress factors in the chicken production environment such as high osmotic stress (dry bedding), low pH (gizzard), anaerobic conditions (caecum), nutrient limitations including iron and high temperature stress (bedding) and, last but not least, stress on *E. coli* from the host defence system. The most serious limitation of the current study is probably that only one strain from each of ST95 and ST131 was studied, and from that, one cannot generalize to common strategies for the two STs. More strains selected with different ecological backgrounds will be needed to draw such conclusions. The estimation of the number of *E. coli* generations in poultry production or any other habitat needs to be improved.

## Supplementary material

10.1099/mgen.0.001893 Supplementary Material 1.

10.1099/mgen.0.001893 Supplementary Material 2.

## Abbreviations

APEC: avian pathogenic *Escherichia coli*
CAI: Codon Adaptation Index
CDSs: coding sequences
COG: cluster of orthologous group
LB: Luria–Bertani
LTEE: long-term evolutionary experiment
ST: sequence type
TSS: transcription start site

## References

[1] Nolan LK, Barnes HJ, Vaillancourt JP, Abdul-Aziz T, Logue CM Diseases of Poultry.

[2] Christensen H, Bachmeier J, Bisgaard M (2021). New strategies to prevent and control avian pathogenic *Escherichia coli* (APEC). Avian Pathol.

[3] Mageiros L, Méric G, Bayliss SC, Pensar J, Pascoe B (2021). Genome evolution and the emergence of pathogenicity in avian *Escherichia coli*. Nat Commun.

[4] Watts A, Wigley P Avian Pathogenic *Escherichia coli*: an overview of infection biology, antimicrobial resistance and vaccination. *Antibiotics*.

[5] Huja S, Oren Y, Trost E, Brzuszkiewicz E, Biran D (2015). Genomic avenue to avian colisepticemia. mBio.

[6] Jørgensen SL, Stegger M, Kudirkiene E, Lilje B, Poulsen LL (2019). Diversity and population overlap between avian and human *Escherichia coli* belonging to sequence type 95. *mSphere*.

[7] Becker S Saidenberg A, van Vliet AHM, Stegger M, Johannesen TB, Semmler T (2022). Genomic analysis of the zoonotic ST73 lineage containing avian and human extraintestinal pathogenic *Escherichia coli* (ExPEC). Vet Microbiol.

[8] Wirth T, Falush D, Lan R, Colles F, Mensa P (2006). Sex and virulence in *Escherichia coli*: an evolutionary perspective. Mol Microbiol.

[9] Jamali H, Akrami F, Bouakkaz S, Dozois CM (2024). Prevalence of specific serogroups, antibiotic resistance and virulence factors of avian pathogenic Escherichia coli (APEC) isolated from clinical cases: a systematic review and meta-analysis. Microb Pathog.

[10] Riley LW (2014). Pandemic lineages of extraintestinal pathogenic *Escherichia coli*. Clin Microbiol Infect.

[11] Pires-dos-Santos T, Bisgaard M, Christensen H (2013). Genetic diversity and virulence profiles of *Escherichia coli* causing salpingitis and peritonitis in broiler breeders. Veterinary Microbiology.

[12] Poulsen LL, Kudirkiene E, Jørgensen SL, Djordjevic SP, Cummins ML (2020). Whole genome sequence comparison of avian pathogenic *Escherichia coli* from acute and chronic salpingitis of egg laying hens. BMC Vet Res.

[13] Zhao Y, Heuvelink A, Olsen JE, Poulsen L, Christensen H (2025). Origin and development of two *Escherichia coli* clones vertically transferred in broiler production. Microb Genom.

[14] Poulsen LL, Thøfner I, Bisgaard M, Christensen JP, Olsen RH (2017). Longitudinal study of transmission of Escherichia *coli* from broiler breeders to broilers. Vet Microbiol.

[15] Lenski RE (2017). Experimental evolution and the dynamics of adaptation and genome evolution in microbial populations. ISME J.

[16] Lenski RE (2023). Revisiting the design of the long-term evolution experiment with *Escherichia coli*. J Mol Evol.

[17] Vasi F, Travisano M, Lenski RE (1994). Long-term experimental evolution in *Escherichia coli*. II. Changes in life-history traits during adaptation to a seasonal environment. The American Naturalist.

[18] Lenski RE, Travisano M (1994). Dynamics of adaptation and diversification: a 10,000-generation experiment with bacterial populations. Proc Natl Acad Sci U S A.

[19] de Visser JAGM, Lenski RE (2002). Long-term experimental evolution in *Escherichia coli*. XI. Rejection of non-transitive interactions as cause of declining rate of adaptation. BMC Evol Biol.

[20] Chen S (2023). Ultrafast one-pass FASTQ data preprocessing, quality control, and deduplication using fastp. *Imeta*.

[21] Deatherage DE, Barrick JE (2014). Identification of mutations in laboratory-evolved microbes from next-generation sequencing data using breseq. Methods Mol Biol.

[22] Nei M, Gojobori T (1986). Simple methods for estimating the numbers of synonymous and nonsynonymous nucleotide substitutions. Mol Biol Evol.

[23] Huerta-Cepas J, Szklarczyk D, Heller D, Hernández-Plaza A, Forslund SK (2019). eggNOG 5.0: a hierarchical, functionally and phylogenetically annotated orthology resource based on 5090 organisms and 2502 viruses. Nucleic Acids Res.

[24] Salamov VSA, Solovyevand A (2011). Automatic annotation of microbial genomes and metagenomic sequences. Metagenomics and its applications in agriculture, biomedicine and environmental studies.

[25] Puigbò P, Bravo IG, Garcia-Vallvé S (2008). E-CAI: a novel server to estimate an expected value of codon adaptation index (eCAI). BMC Bioinformatics.

[26] Mirdita M, Schütze K, Moriwaki Y, Heo L, Ovchinnikov S (2022). ColabFold: making protein folding accessible to all. Nat Methods.

[27] Meng EC, Goddard TD, Pettersen EF, Couch GS, Pearson ZJ (2023). UCSF ChimeraX: Tools for structure building and analysis. Protein Sci.

[28] Chen Y, Lu H, Zhang N, Zhu Z, Wang S (2020). PremPS: predicting the impact of missense mutations on protein stability. PLoS Comput Biol.

[29] Nielsen SS, Alvarez J, Bicout DJ, Calistri P, Canali E (2023). Welfare of broilers on farm. EFS2.

[30] Barrick JE, Blount ZD, Lake DM, Dwenger JH, Chavarria-Palma JE (2023). Daily Transfers, archiving populations, and measuring fitness in the long-term evolution experiment with *Escherichia coli*. J Vis Exp.

[31] Bougdour A, Wickner S, Gottesman S (2006). Modulating RssB activity: IraP, a novel regulator of sigma(S) stability in *Escherichia coli*. Genes Dev.

[32] Micevski D, Zammit JE, Truscott KN, Dougan DA (2015). Anti-adaptors use distinct modes of binding to inhibit the RssB-dependent turnover of RpoS (σ(S)) by ClpXP. Front Mol Biosci.

[33] Schellhorn HE (2020). Function, evolution, and composition of the RpoS regulon in *Escherichia coli*. Front Microbiol.

[34] King T, Ishihama A, Kori A, Ferenci T (2004). A regulatory trade-off as a source of strain variation in the species *Escherichia coli*. J Bacteriol.

[35] Bougdour A, Gottesman S (2007). ppGpp regulation of RpoS degradation via anti-adaptor protein IraP. Proc Natl Acad Sci U S A.

[36] Bontemps-Gallo S, Cogez V, Robbe-Masselot C, Quintard K, Dondeyne J (2013). Biosynthesis of osmoregulated periplasmic glucans in Escherichia coli: the phosphoethanolamine transferase is encoded by opgE. Biomed Res Int.

[37] Bondage DD, Lin JS, Ma LS, Kuo CH, Lai EM (2016). VgrG C terminus confers the type VI effector transport specificity and is required for binding with PAAR and adaptor-effector complex. Proc Natl Acad Sci U S A.

[38] Alves JA, Leal FC, Previato-Mello M, da Silva Neto JF (2022). A quorum sensing-regulated type VI secretion system containing multiple nonredundant VgrG proteins is required for interbacterial competition in chromobacterium violaceum. Microbiol Spectr.

[39] Remold SK, Lenski RE (2001). Contribution of individual random mutations to genotype-by-environment interactions in *Escherichia coli*. Proc Natl Acad Sci U S A.

[40] Couce A, Limdi A, Magnan M, Owen SV, Herren CM (2024). Changing fitness effects of mutations through long-term bacterial evolution. Science.

[41] Barrick JE, Yu DS, Yoon SH, Jeong H, Oh TK (2009). Genome evolution and adaptation in a long-term experiment with *Escherichia coli*. Nature.

[42] Tenaillon O, Barrick JE, Ribeck N, Deatherage DE, Blanchard JL (2016). Tempo and mode of genome evolution in a 50,000-generation experiment. Nature.

[43] Maharjan RP, Ferenci T (2017). A shifting mutational landscape in 6 nutritional states: Stress-induced mutagenesis as a series of distinct stress input-mutation output relationships. PLoS Biol.

[44] Phan K, Ferenci T (2013). A design-constraint trade-off underpins the diversity in ecologically important traits in species *Escherichia coli*. ISME J.

[45] Bouma JE, Lenski RE (1988). Evolution of a bacteria/plasmid association. Nature.

[46] Khan AI, Dinh DM, Schneider D, Lenski RE, Cooper TF (2011). Negative epistasis between beneficial mutations in an evolving bacterial population. Science.

[47] Lee H, Popodi E, Tang H, Foster PL (2012). Rate and molecular spectrum of spontaneous mutations in the bacterium *Escherichia coli* as determined by whole-genome sequencing. Proc Natl Acad Sci U S A.

[48] Liu Y, Yang Q, Zhao F (2021). Synonymous but Not Silent: The Codon Usage Code for Gene Expression and Protein Folding. Annu Rev Biochem.

