# Supplementary material for: Evolutionary dynamics and differential mutation strategies of two avian pathogenic Escherichia coli lineages during experimental evolution
Source: Microb Genom. 2026 Nov 13;12(11):001893. doi: 10.1099/mgen.0.001893 (PMC13766299; doi:10.1099/mgen.0.001893)

Supplementary material for the paper

Evolutionary dynamics and differential mutation strategies of two avian pathogenic *Escherichia coli* lineages during experimental evolution

By Yufei Zhao, Luo Yang, Dejun Liu, Jing Li and Henrik Christensen[1*]

Supplementary Data Section

Assembly accession number	WGS project accession number	Strain
GCA_055687555.1	JBVPTT000000000	729-021014-1-4_1.19_1
GCA_055687525.1	JBVPTS000000000	729-021014-1-4_1.19_2
GCA_055687505.1	JBVPTR000000000	729-021014-1-4_1.19_3
GCA_055687485.1	JBVPTQ000000000	729-021014-1-4_1.26_1
GCA_055687465.1	JBVPTP000000000	729-021014-1-4_1.26_2
GCA_055687445.1	JBVPTO000000000	729-021014-1-4_1.26_3
GCA_055687425.1	JBVPTN000000000	729-021014-1-4_2.1_1
GCA_055687405.1	JBVPTM000000000	729-021014-1-4_2.1_2
GCA_055687385.1	JBVPTL000000000	729-021014-1-4_2.1_3
GCA_055687365.1	JBVPTK000000000	729-021014-1-4_2.8_1
GCA_055687345.1	JBVPTJ000000000	729-021014-1-4_2.8_2
GCA_055687325.1	JBVPTI000000000	729-021014-1-4_2.8_3
GCA_055687305.1	JBVPTG000000000	729-021014-1-4_2.15_2
GCA_055687285.1	JBVPTH000000000	729-021014-1-4_2.15_1
GCA_055687265.1	JBVPTF000000000	729-021014-1-4_2.15_3
GCA_055687245.1	JBVPTE000000000	729-021014-1-4_2.22_1_
GCA_055687225.1	JBVPTD000000000	729-021014-1-4_2.22_2
GCA_055687205.1	JBVPTC000000000	729-021014-1-4_2.22_3
GCA_055687185.1	JBVPTA000000000	729-021014-1-4_2.29_1
GCA_055687165.1	JBVPTB000000000	729-021014-1-4_2.22_4
GCA_055687145.1	JBVPSZ000000000	729-021014-1-4_2.29_2
GCA_055687125.1	JBVPSY000000000	729-021014-1-4_2.29_3
GCA_055687105.1	JBVPSX000000000	729-021014-1-4_2.29_4
GCA_055687085.1	JBVPSW000000000	729-021014-1-4_2.29_5
GCA_055687065.1	JBVPSU000000000	729-021014-1-4_3.7_2
GCA_055687045.1	JBVPSV000000000	729-021014-1-4_3.7_1
GCA_055687025.1	JBVPST000000000	729-021014-1-4_3.7_3
GCA_055686965.1	JBVPSQ000000000	729-021014-1-4_3.7_6
GCA_055687005.1	JBVPSS000000000	729-021014-1-4_3.7_4
GCA_055686985.1	JBVPSR000000000	729-021014-1-4_3.7_5
GCA_055686945.1	JBVPSP000000000	729-021014-1-4_3.7_7
GCA_055686925.1	JBVPSO000000000	729-021014-1-4_3.7_8
GCA_055686905.1	JBVPSN000000000	729-141114-1-15_6.5_1
GCA_055686885.1	JBVPSM000000000	729-141114-1-15_6.5_2
GCA_055686865.1	JBVPSL000000000	729-141114-1-15_6.5_3
GCA_055686845.1	JBVPSK000000000	729-141114-1-15_6.12_1
GCA_055686825.1	JBVPSJ000000000	729-141114-1-15_6.12_2
GCA_055686805.1	JBVPSI000000000	729-141114-1-15_6.12_3
GCA_055686785.1	JBVPSH000000000	729-141114-1-15_6.19_1
GCA_055686765.1	JBVPSG000000000	729-141114-1-15_6.19_2
GCA_055686745.1	JBVPSF000000000	729-141114-1-15_6.19_3
GCA_055686725.1	JBVPSE000000000	729-141114-1-15_6.26_1
GCA_055686705.1	JBVPSD000000000	729-141114-1-15_6.26_2
GCA_055686685.1	JBVPSC000000000	729-141114-1-15_6.26_3
GCA_055686665.1	JBVPSB000000000	729-141114-1-15_7.3_1
GCA_055686645.1	JBVPSA000000000	729-141114-1-15_7.3_2
GCA_055686625.1	JBVPRZ000000000	729-141114-1-15_7.3_3
GCA_055686595.1	JBVPRY000000000	729-141114-1-15_7.10_1
GCA_055686585.1	JBVPRW000000000	729-141114-1-15_7.10_3
GCA_055686565.1	JBVPRX000000000	729-141114-1-15_7.10_2
GCA_055686545.1	JBVPRV000000000	729-141114-1-15_7.17_1
GCA_055686525.1	JBVPRT000000000	729-141114-1-15_7.17_3
GCA_055686505.1	JBVPRU000000000	729-141114-1-15_7.17_2
GCA_055686485.1	JBVPRS000000000	729-141114-1-15_7.24_1
GCA_055686465.1	JBVPRR000000000	729-141114-1-15_7.24_2
GCA_055686445.1	JBVPRQ000000000	729-141114-1-15_7.24_3

Supplementary Fig. S1. The protein structure of *klcA non-synonoymous* mutation before (grey) and after (red) evolution. In zoom in view the mutation caused an increase from 1 atom on the side chain to 3 atoms after evolution..

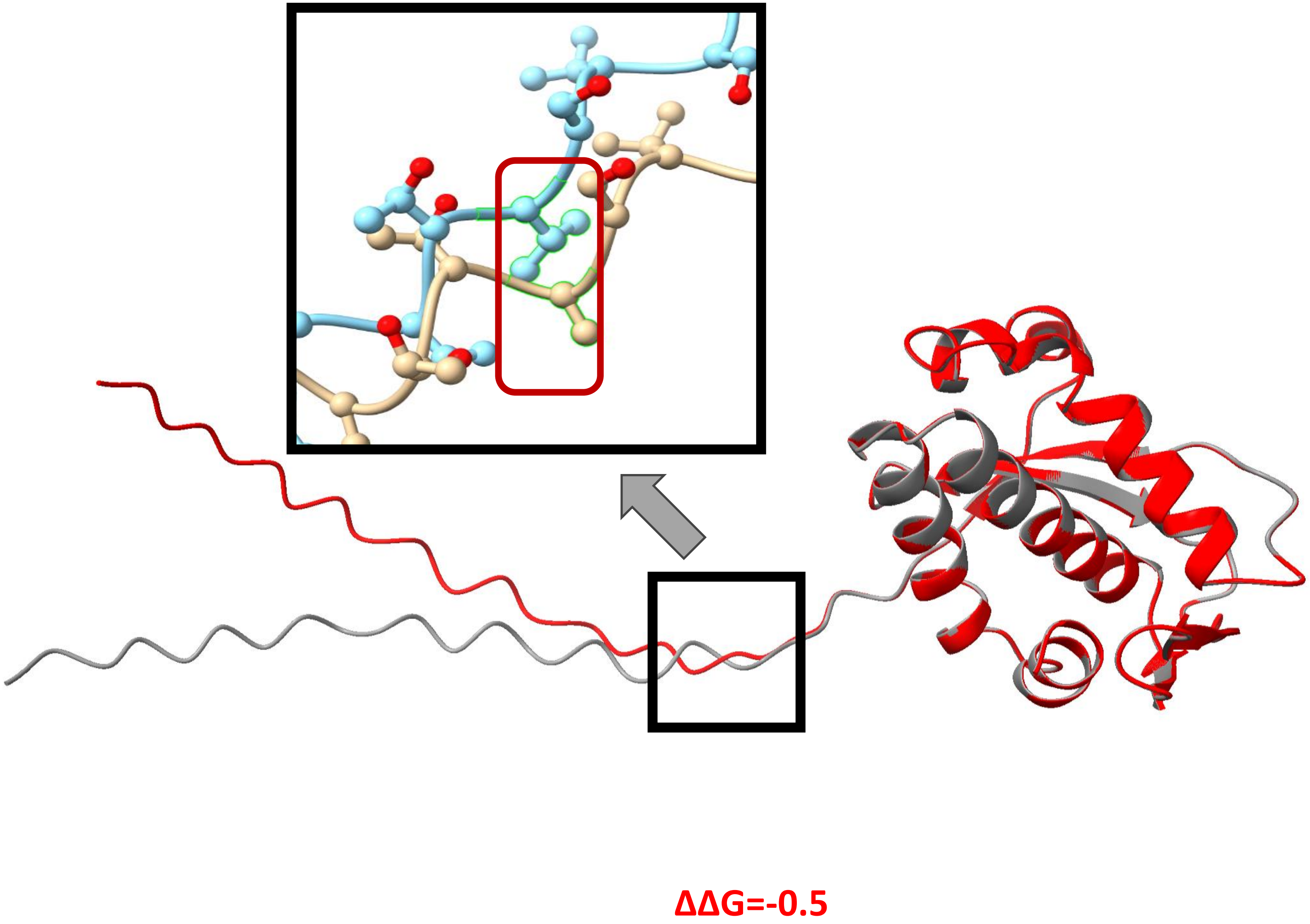

Supplement: Supplementary file 1 [file mgen-12-01893-s001.pdf]
